# Accelerated Left-Handed DNA-PAINT Using Fluorogenic Probes

**DOI:** 10.1021/acs.nanolett.5c05973

**Published:** 2026-04-29

**Authors:** Bas van Bommel, Helge Ewers

**Affiliations:** † Institut für Biochemie, 54203 Freie Universität Berlin, Thielallee 63, 14195 Berlin, Germany

**Keywords:** Single-molecule localization microscopy, Super-resolution microscopy, DNA-PAINT, Fluorogenic DNA-PAINT, Fluorogenic left-handed DNA-PAINT, Volumetric DNA-PAINT

## Abstract

Single-molecule localization microscopy (SMLM) techniques offer nanometer-scale resolution by stochastically localizing individual fluorescent molecules. Among these, DNA-mediated point accumulation for imaging in nanoscale topography (DNA-PAINT) stands out for its high multiplexing capability and excellent localization accuracy. In this work, we introduce a fast method using left-handed DNA (L-DNA) probes that show a lower background in DNA-PAINT experiments. The use of L-DNA reduces the background originating from unspecific imager and antibody binding. When fluorogenic probes are employed, emission from free binders is quenched, opening access to volumetric DNA-PAINT. Additionally, like fluorogenic right-handed (R-DNA-)­PAINT, fluorogenic L-DNA-PAINT allows for lower integration times and faster accumulation of localizations. The binding kinetics, brightness, and localization precision of the fluorogenic R- and L-DNA probes are indistinguishable. Furthermore, fluorogenic left- and right-handed probes do not show cross-reactivity, thus expanding the range of available sequences for reliable multiplexed imaging. Integrating L-DNA-PAINT with a fluorogenic probe design significantly expands the experimental versatility of DNA-PAINT.

## Graphical abstract

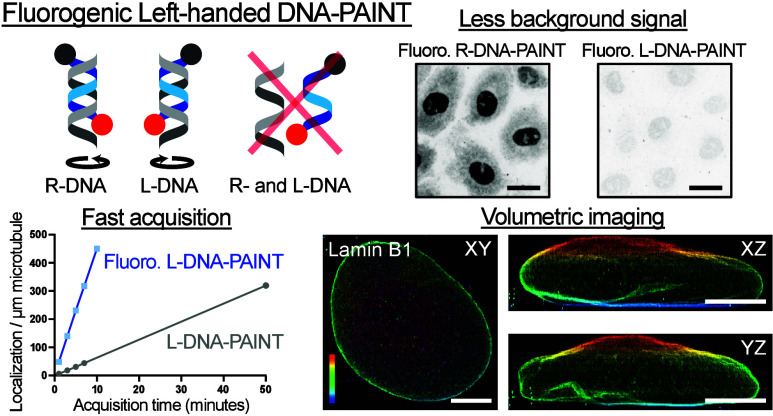

Single-molecule localization microscopy (SMLM) has become a powerful technique for obtaining quantitative information from biological samples. By fitting the point spread function of individual fluorophores and reconstructing images from these localizations, SMLM enables microscopy with an accuracy that surpasses the diffraction limit.^1–3^ To extract information from complex samples containing many fluorophores, various strategies have been developed to temporally separate emission events, thereby allowing reliable single-molecule fitting. These approaches range from the use of photoswitchable proteins to direct stochastic optical reconstruction microscopy (dSTORM)^4^ and point accumulation for imaging in nanoscale topography (PAINT).^5^

PAINT can be realized through DNA hybridization, in which short, fluorophore-labeled “imager” strands transiently bind to their complementary docking strands.^6,7^ This transient binding immobilizes the fluorophore briefly, producing a bright localization signal on the detector. DNA-PAINT offers several key advantages: the use of bright synthetic fluorophores yields high photon counts and thus precise fitting; the continuous exchange of imager strands minimizes the effects of photobleaching, allowing prolonged imaging time and stringent localization selection; the imager concentration can be tuned to control the localization density within a given time window. Furthermore, the vast sequence diversity of DNA enables multiplexed imaging of targets within the same sample,^7–9^ and the excellent control over kinetics allows for quantitative imaging.^10^ Together, these features make DNA-PAINT a uniquely versatile super-resolution method capable of achieving nanoscale resolution.^11^

In DNA-PAINT, the balance between signal generation and background suppression is critical for achieving an efficient and precise image reconstruction. Optimal image reconstruction requires carefully tuned DNA hybridization kinetics, while the nonspecific binding of imager strands in the sample must be minimized. The background can be reduced through appropriate titration of the imager strand concentration and illumination strategies such as total internal reflection fluorescence (TIRF) microscopy.^12,13^ Additional improvements can be achieved by employing L-DNA, a left-handed DNA analogue with reduced affinity for endogenous nucleic acids, which is particularly advantageous for imaging near or within the nucleus.^14^ Other recent advances have further improved DNA-PAINT by accelerating hybridization kinetics, enabling faster acquisition of data for multiple targets.^15–17^ This significantly improves the throughput of such experiments, reduces drift influences, and generally improves the reconstruction quality.

Here we aimed to accelerate the data acquisition speed and allow volumetric imaging for L-DNA-PAINT by introducing a fluorogenic probe design. The use of fluorogenic probes allows for faster image acquisition compared to DNA-PAINT using nonfluorogenic probes and buffers.^18^ The fluorogenic properties of the probes are maintained when using DNA with a left-circulating backbone. The binding time, brightness, and localization accuracy are on par with its R-DNA counterpart. The minimal to absent hybridization of L-DNA to right-handed DNA (R-DNA) reduces the background occurring from unspecific binding of both imager strands and, importantly, also antibody–docking strand conjugates. Moreover, this element can be further used for multiplexing in DNA-PAINT. By combining fluorogenic L- and R-DNA probes, it is possible to image multiple targets using identical DNA base-pair combinations. The use of fluorogenic L-DNA probes widens the DNA-PAINT toolbox and improves quality, especially acquisitions deeper in the cells.

## Accelerating L-DNA-PAINT

We first aimed to test whether L-DNA imager strands would exhibit fluorogenic properties upon binding when prepared in a conformation with a dye–quencher pair, as reported for R-DNA.^16^ To do so, we used a published sequence and directly compared R- and L-DNA using the same fluorophore–quencher pair (F1R/F1L) (Figure 1a and Supporting Figure 1a). The corresponding R- and L-DNA docking strands were conjugated to secondary antibodies. Using this configuration, we performed immunocytochemistry for microtubules in COS-7 cells. Microtubules are established as a reference target for super-resolution microscopy because their structure is well-known and their super-resolved image allows for quantitative quality control.^19,20^ When we then performed DNA-PAINT using these oligomers, we obtained high-quality reconstructions, demonstrating that fluorogenic L-DNA probes work effectively (Figure 1a and Supporting Figure 1a). A close look at individual microtubules shows that the tubules are resolved at high resolution (insets). Additional spectral analysis of the fluorogenic probes shows that both R- and L-DNA imager strands display fluorogenic properties (Figure 1b). The fluorogenic L-DNA imager strands show slightly higher emission in the unbound state compared to their R-DNA counterparts, which could be due to differences in the synthesis efficiency for attachment of the quencher to the 3′ end of the imager. Because L-DNA only differs from R-DNA in the chiral orientation of the DNA backbone, it is unlikely that this difference is sequence-related. Upon binding, the emission peaks of both R- and L-DNA probes show a strong increase, illustrating the fluorogenic properties (fluorogenic R-DNA-PAINT unbound peak value 2.4% versus bound imager and fluorogenic L-DNA-PAINT unbound peak value 16.1% versus bound imager). Interestingly, we also observed a strong change in absorption between the unbound and bound states of the probes. Upon binding and stretching of the imager strands, when the quencher is spaced away from the fluorophore, the observed absorption spectrum shifts toward the known absorption spectrum of the fluorophore. In general, we observed that when fluorogenic probes were bound, the absorption and emission spectra were very similar to those of the (unquenched) fluorophores (Supporting Figure 1c). The fluorogenic probe designs permit a higher DNA imager concentration added to the sample and increased excitation power and, together with the improved binding kinetics, allow for faster data acquisition. We compared the performance of this new fluorogenic L-DNA probe to nonfluorogenic L-DNA-PAINT imager sequences as we used them before.^14^ When we analyzed the accumulation of localizations over time, we observed that many more localizations could be obtained in a smaller time window with the fluorogenic L-DNA probe in comparison with the standard configuration (Figure 1c). A comparable image quality could be reconstructed in 10 min compared to 50 min using nonfluorogenic probes, illustrating the speed gain that comes with the use of fluorogenic probes.

**Figure 1:**
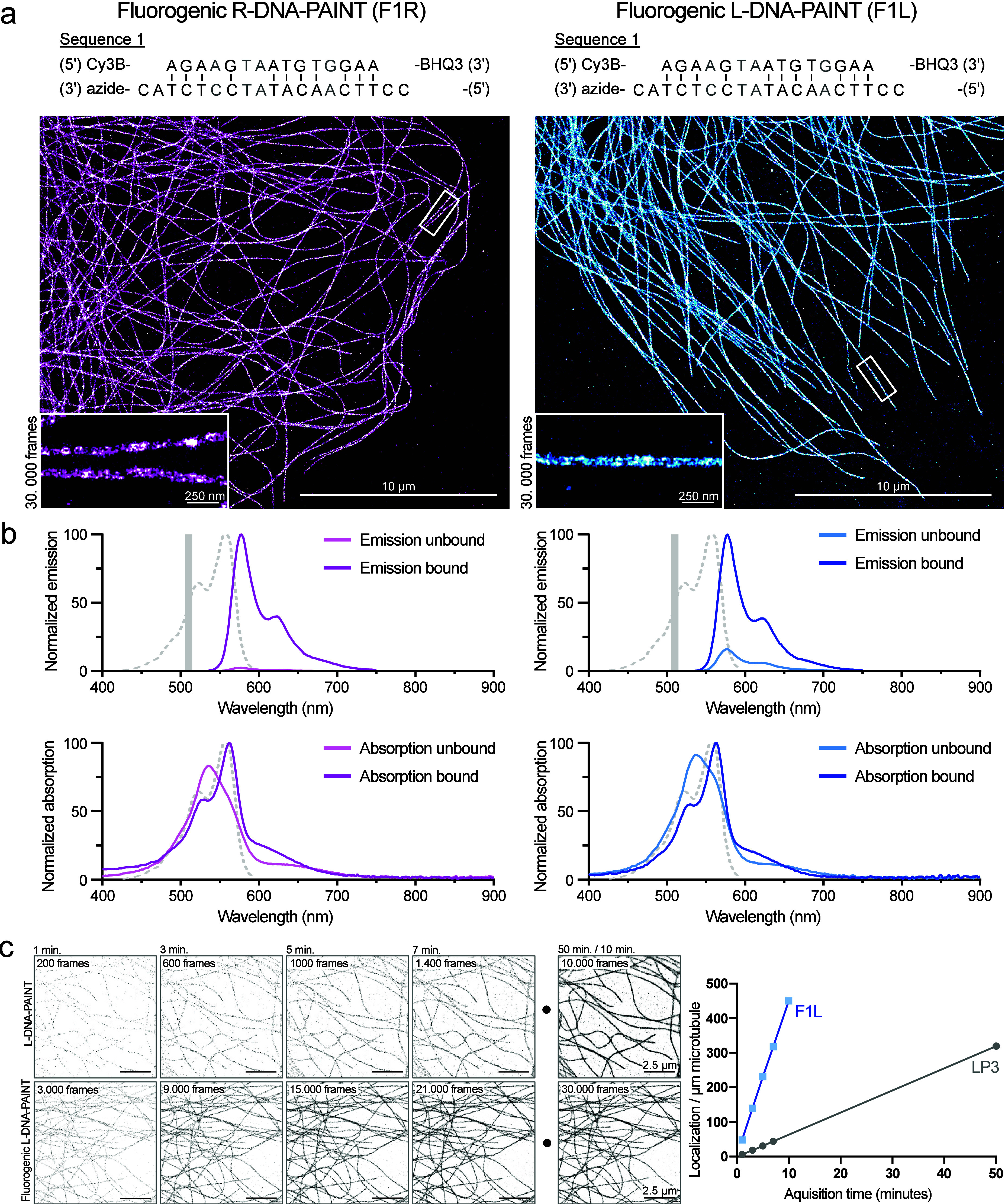
Accelerating L-DNA-PAINT. (a) Examples of fluorogenic R- and L-DNA-PAINT of microtubules in COS-7 cells acquired in TIRF. Microtubules are consistently labeled; high resolution can be achieved at crossings, and insets show detailed nanostructure information. (b) Normalized emission (top) and absorption (bottom) spectra of unbound and bound fluorogenic DNA-PAINT probes recorded with a plate reader. Both fluorogenic R- and L-DNA-PAINT imager strands show a strong increase of emission in the bound condition. Absorption spectra also show a shift between unbound and bound states. The solid gray line indicates the excitation light. The dotted light-gray line indicates the emission/absorption of Cy3B as a single dye (data from fpbase.org^200^). (c) Time-lapse series of L-DNA-PAINT with nonfluorogenic (1 nM imager strand, 300 ms exposure) and fluorogenic (10 nM imager strand, 20 ms exposure) probe design were recorded in TIRF. Fluorogenic L-DNA probes significantly shorten the acquisition time, from 50 to 10 min in this particular example. Examples are scaled with identical brightness and contrast settings. Localizations per micron microtubule were estimated from the displayed images.

## Fluorogenic L-DNA-PAINT Offers Excellent Precision

For reliable statistics on resolution, we then performed multiple acquisitions of microtubules (as depicted in Figure 2a). The distribution of localization precision estimated by the Cramér-Rao Lower Bound, as a first indicator, showed little to no difference between fluorogenic R- and L-DNA probes of the same sequence [two-way repeated-measures ANOVA with Idák’s correction for multiple comparison: n.s. except p (5 nm bin) = 0.01] (Figure 2b and Supporting Figure 2a). This can also be estimated from localization precision determined by the nearest-neighbor estimate (Supporting Figure 2b).^21^ Similarly, the probes exhibit very comparable binding times and photon counts (Supporting Figure 3).

**Figure 2:**
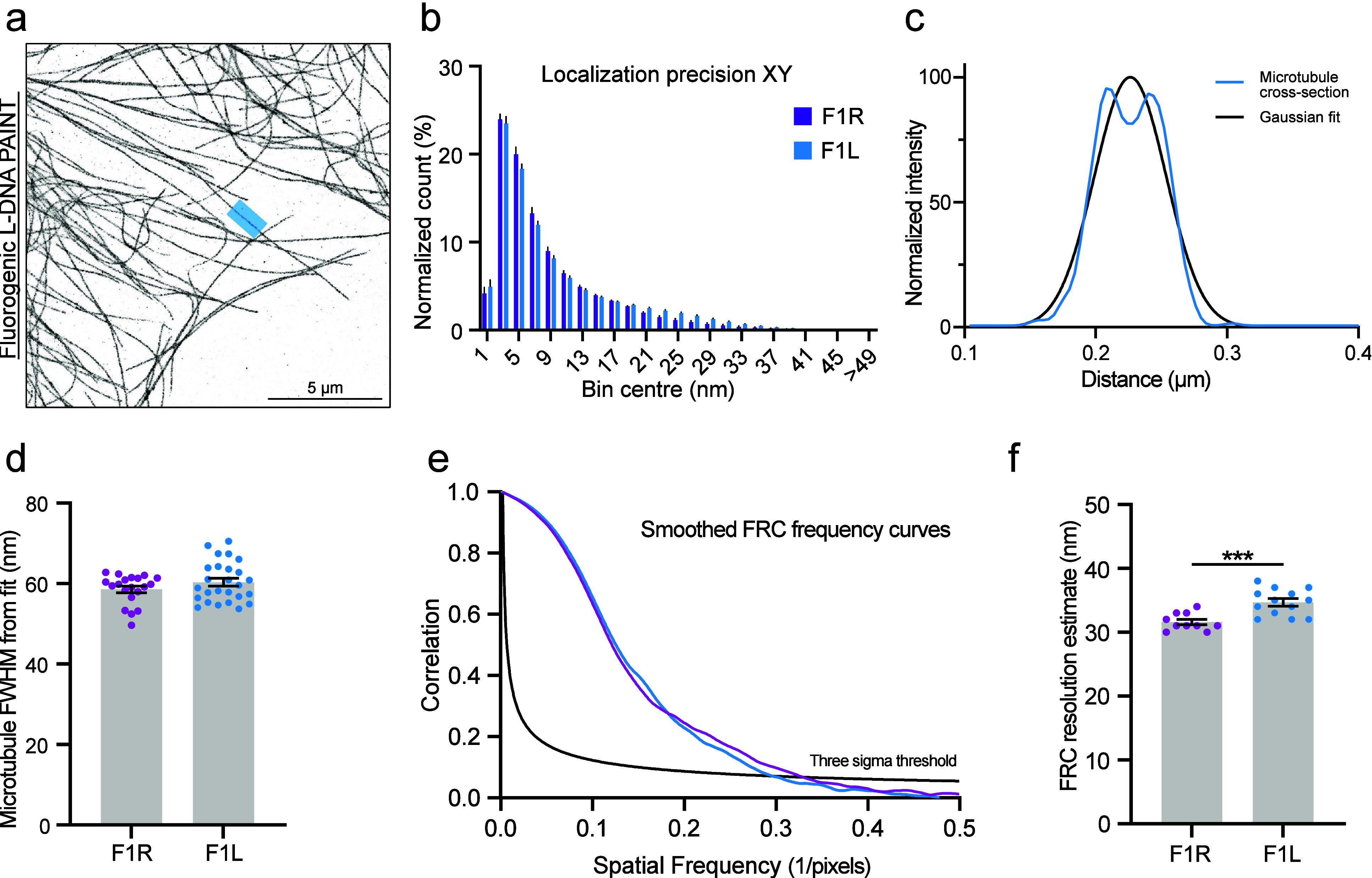
Fluorogenic L-DNA-PAINT offers excellent precision. (a) Example of microtubules recorded with fluorogenic L-DNA-PAINT probes in TIRF. The blue region indicates the plotted section in part c. (b) Distribution of the Cramér-Rao Lower Bound for fluorogenic R- and L-DNA-PAINT in *XY*. The estimated localization precision for the two probes is highly similar. Bars show the average of multiple acquisitions with SEM. Error bars are plotted upward only (repeated-measures two-way ANOVA with Šidák’s test for multiple comparisons, n.s. except for 5 nm bin: *p* = 0.01). (c) Plot profile of the microtubule indicated in part a (blue). The two peaks correspond to the left and right sides of the microtubule. Gaussian fit to estimate the FWHM as a measure for the microtubule diameter (black). (d) Quantification of the microtubule diameter by the FWHM of the Gaussian fit. Two microtubule segments were measured per acquisition. Error bars show SEM. The measured FWHM of microtubules is indistinguishable between fluorogenic R- and L-DNA-PAINT probes. Bars show the average with SEM (two-tailed unpaired Student’s *t* test, difference between means 1.8 nm, *p* = 0.19). (e) Example of a FRC measurement for fluorogenic R- and L-DNA-PAINT acquisition. (f) Quantification of FRC for multiple fluorogenic R- and L-DNA-PAINT acquisitions. The difference in the mean is small. Bars show the average of multiple acquisitions with SEM (two-tailed unpaired Student’s *t* test, difference between means 3.1 nm, *p* < 0.001).

We next estimated the quality of the data by measuring the full width at half-maximum (FWHM) of microtubules and the Fourier ring correlation (FRC) from reconstructions. Microtubule cross-sectional profiles often showed two small peaks representing the sides of the tube structure. Fitting a Gaussian distribution on the cross profile allows measurement of the microtubule FWHM (Figure 2c). Analysis of 20 (R-DNA) and 26 (L-DNA) microtubules (2 per acquisition) shows no difference in the microtubule width between fluorogenic R- and L-DNA-PAINT (two-tailed unpaired *t* test, *p* = 0.19) (Figure 2d and Supporting Figure 2c). For estimating the overall resolution of the acquisitions, we split the localizations of one acquisition into two groups in order to create two images for measuring the FRC (Figure 2e,f and Supporting Figure 2d).^22^ A small difference was observed between the fluorogenic probes. However, this small difference likely originates from factors such as drift during acquisitions and density differences between samples (more dense or sparse microtubules) and was therefore considered negligible. Our overall measured resolution parameters suggest very high imaging quality, comparable to earlier reports in the literature.^19,23^ Taken together, we found that fluorogenic R- and L-DNA-PAINT probes, regardless of their different backbones, behave very similarly. They can both be used to achieve high-quality nanoscale reconstructions at high precision.

## Fluorogenic L-DNA-PAINT Significantly Reduces Background Binding

We next asked whether the reported reduction in the background binding of L-DNA compared to R-DNA could also be observed for the more transiently binding fluorogenic L-DNA imager strands. We found that fluorogenic L-DNA-PAINT reduces the background to a minimum in the cytoplasm and especially in the nucleus (Figure 3). In fact, in samples with unlabeled cells incubated with only imager strands, the nucleus was clearly visible for fluorogenic R-DNA imagers due to unspecific binding, even including morphological detail. On the other hand, when we added fluorogenic L-DNA imagers to the same cells, the nuclei could not be distinguished from the background (Figure 3a and Supporting Figure 4a). Further supporting this observation, when we quantified the binding times of these unspecific events, signals in the fluorogenic L-DNA-PAINT acquisitions were mostly short-lived events (1 frame, 20 ms), originating from pixel noise or transient, unproductive binding which can be routinely removed by increasing the detection threshold. In contrast, fluorogenic R-DNA-PAINT probes exhibited a significantly higher fraction of longer binding events, suggesting at least occasional bona fide hybridization instead (Figure 3b, binding times background: two-way repeated measures ANOVA with Šidák’s test for multiple comparisons: 20–60 ms,*p* < 0.001; 80 ms, *p* = 0.04).

**Figure 3:**
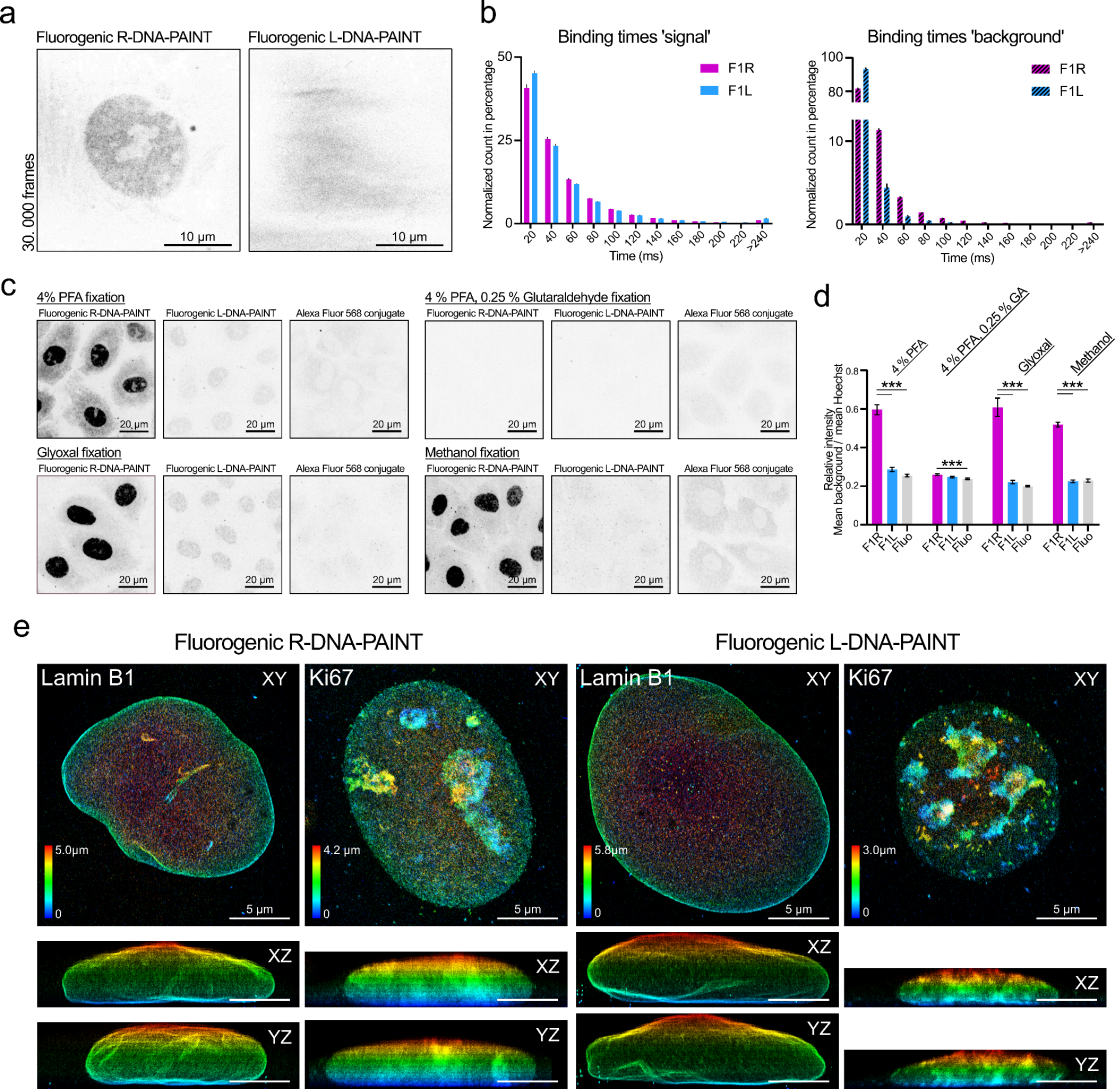
Fluorogenic L-DNA-PAINT reduces the background and improves volumetric imaging. (a) Examples of background patterns resulting from unspecific binding of the imager probes recorded with HILO excitation. Fluorogenic R-DNA-PAINT shows background binding in the nucleus, while fluorogenic L-DNA-PAINT probes solely show illumination/camera noise. (b) Left: Binding times recorded from microtubule samples. Data were recorded with 20 ms exposure time per frame. Right: Binding times of noise patterns, as illustrated in part a. Fluorogenic L-DNA-PAINT shows fewer events that persist across multiple frames. Bars show the average of multiple acquisitions with SEM. Error bars are plotted upward only (Binding times “signal”: repeated-measures two-way ANOVA with Šidák’s test: 20–40 ms, *p* < 0.001; 60 ms, *p* = 0.02. Binding times “background”: repeated-measures two-way ANOVA with Šidák’s test: 20–60 ms, *p* < 0.001; 80 ms, *p* = 0.04). (c) Confocal maximum intensity projections showing unspecific binding of conjugated secondary antibodies in different fixation protocols. Secondary antibodies were conjugated with F1R or F1L docking strands or with Alexa Fluor 568. Unspecific binding was detected with an antigoat Alexa Fluor 647 antibody. (d) Quantification of background binding in the nucleus, as shown in part c. The background is significantly higher for fluorogenic R-DNA antibody conjugates in PFA, glyoxal, and methanol fixed cells (one-way ANOVA with Tukey’s multiple comparison test: PFA, glyoxal, and methanol fixation, F1R vs F1L and F1R vs Alexa Fluor 568, *p* < 0.001; PFA/GA fixation, F1R vs Alexa Fluor 568, *p* = 0.001; other comparisons show no significant differences). (e) Volumetric fluorogenic R- and L-DNA-PAINT of Lamin B1 and Ki67 acquired in biplane mode with wide-field illumination. Acquired with a step size of 0.5 μm, from bottom upward until the nuclear volume was covered. Accumulated Gaussian reconstructions are color-coded for the *Z* axis.

In a second experiment, we tested whether the secondary antibody–docking DNA conjugates would equally display unspecific binding and have the potential to cause background (as reported by Lučinskaitė et al.^24^). To do this, we incubated cells, prepared with different fixation methods, directly with DNA docking strand coupled goat secondary antibodies (Figure 3c). No primary antibody for a cellular protein/target was present. We next applied an antigoat polyclonal Alexa Fluor 647 antibody. This amplifies, due to its polyclonal composition, any unspecifically bound secondary antibody and provides a sensitive assay for the quantification of unspecific binding. We found that antibodies conjugated to R-DNA docking strands showed strong unspecific binding in PFA, glyoxal, and methanol fixed samples (Figure 3c). This background binding was significantly reduced when identical secondary antibodies were conjugated with L-DNA docking strands (*p* < 0.001, one-way ANOVA with Tukey’s multiple comparison for each fixation method) (Figure 3d). Fixation with PFA in combination with glutaraldehyde showed surprisingly less background for both R- and L-DNA-PAINT probes. While glutaraldehyde fixation can be excellent for some targets (microtubules and membrane proteins), it also has the potential to mask epitopes for antibody binding. These results show that the off-target/background binding for DNA-conjugated antibodies can be reduced by using L-DNA. This effect cannot be fully attributed to unspecific DNA–DNA hybridization only. The off-target signal may also originate from R-DNA interactions with nuclear proteins. In summary, the background signal in fluorogenic DNA-PAINT can be reduced by using L-DNA, where the benefits originate from reduced interactions of both the imager and docking strand conjugated antibodies with the sample.

Fluorogenic left-handed probe design combines the advantages of fluorogenic probes with L-DNA, resulting in fast acquisition and a lower background. This opens up possibilities for volumetric DNA-PAINT of the cells. We tested this for two nuclear targets, nuclear-membrane-bound Lamin B1 and Ki67, a marker for DNA replication sites (Figure 3e and Supporting Figure 5). We acquired the total volume of nuclei in wide-field illumination in combination with z-stepping in the biplane mode. Fluorogenic R- and L-DNA probes worked for volumetric imaging, but with L-DNA, we generally obtained more contrasted images. This is particularly visible in the accumulated Gaussian side projections, in which the whole nuclear volume is compressed along the axis. We concluded that fluorogenic L-DNA-PAINT can be advantageous for volumetric DNA-PAINT, especially when the sampled volume contains nuclei.

## L-DNA-PAINT for Multiplexed Microscopy

In a next set of experiments, we exploited the unique feature of L-DNA: its minimal hybridization with R-DNA. This allows the acquisitions of two targets with DNA probes of the identical sequence, once in R-DNA and once in L-DNA. When we labeled microtubules with an additional monoclonal antibody recognizing acetyltubulin, a post-translational modification typically occurring on the inside of microtubules,^20^ both confocal images (as references) and SMLM reconstructions showed that some microtubules were strongly acetylated and others to a lesser extent^25^ (Figure 4a–c). These reconstructions were obtained from sequential imaging of the samples with fluorogenic R- and L-DNA probes for total and acetylated tubulin, respectively. The two populations of microtubules are also represented in Pearson’s pixel-based cross correlation, by a density cloud mostly present at the *x* axis (α/β-tubulin), which extends toward the *y* axis (acetyl. tub.) (Figure 4c).

**Figure 4:**
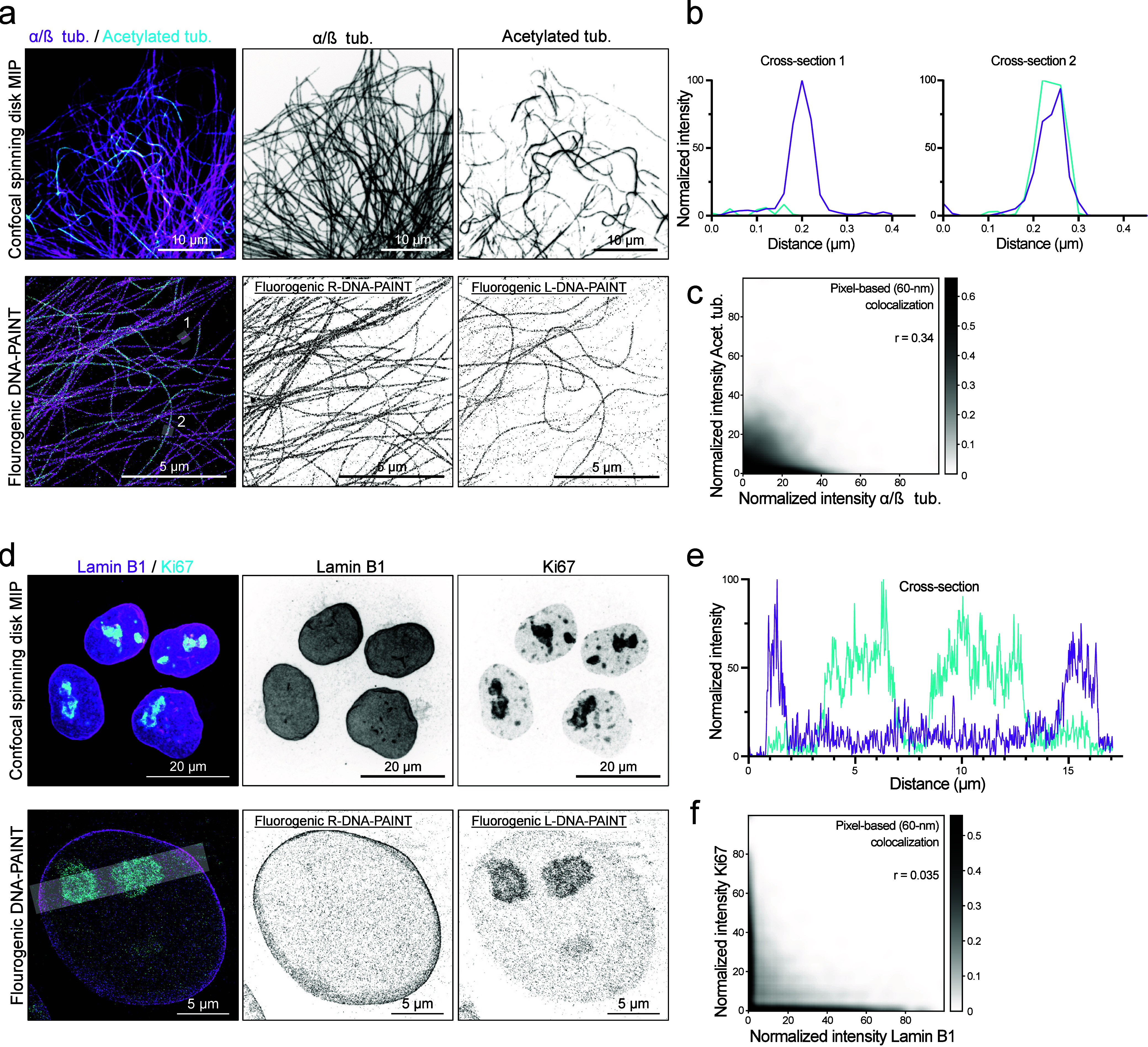
Fluorogenic L-DNA-PAINT for multiplexed imaging. (a) Examples of dual-channel acquisitions of microtubules and acetylated microtubules. Top: Maximum intensity projection of a confocal *Z*-stack. Bottom: Example of a fluorogenic R- and L-DNA-PAINT acquisition. (b) Cross-sectional intensity profiles from the segments indicated in part a. Section 1 of a nonacetylated microtubule and section 2 from a heavily acetylated microtubule. (c) Pixel-based Pearson’s cross-correlation graph from the DNA-PAINT acquisitions presented in part a. The highest localization density is found for the α/β tub. channel. A proportion of the microtubules is acetylated, which is reflected as an upward bias of the density cloud. (d) Examples of dual-channel acquisitions of Lamin B1 and Ki67. Top: Maximum intensity projection of a confocal *Z*-stack. Bottom: Example of a fluorogenic R- and L-DNA-PAINT sequential acquisition with an identical base-pair sequence and fluorophore for both cellular targets. (e) Intensity profile along the section indicated in part d. Peaks of the individual channels do not match, indicating that there is no cross-hybridization between fluorogenic R- and L-DNA-PAINT probes. (f) Pixel-based Pearson’s cross-correlation graph from the DNA-PAINT acquisitions presented in part d. Fluorogenic R-DNA-PAINT (Lamin B1) and L-DNA-PAINT (Ki67) probes show distinct independent localizations, represented as a separated density profile along the *X* and *Y* axes, indicating no cross-hybridization between fluorogenic R- and L-DNA-PAINT probes.

In addition to microtubules, we also targeted a more challenging cellular component, the nucleus. We stained the nuclear membrane with an anti-Lamin B1 polyclonal antibody and R-DNA probes and Ki67, a protein enriched at sites of DNA replication, with a monoclonal antibody and L-DNA probes (Figure 4d). These targets lie deeper within the cells; therefore, the detection threshold for localizations was increased to minimize the contribution of noise. This resulted in a lower number of localizations, but allowed for high-quality reconstructions. Plotting a cross section through the nucleus and DNA replication sites shows that there is minimal to no cross binding between fluorogenic R- and L-DNA probes (Figure 4e). This is equally visible from the pixel-based Pearson’s cross-correlation, which is clearly defined by densities around both the *X* and *Y* axes (Figure 4f). This experiment shows that fluorogenic R- and L-DNA probes with identical DNA sequence can be used for sequential multiplexing, thus doubling the number of available fluorogenic probes for multiplexed nanoscale microscopy.

We here demonstrate that probe design using dye-quencher pairs at the 3′ and 5′ termini of DNA-imager strands for DNA-PAINT, which yield fast data acquisitions and high-quality reconstructions, is directly transferable to L-DNA. We find that fluorogenic L-DNA-PAINT yields high-quality nanoscale data with minimal background and a resolution comparable to that of R-DNA-based fluorogenic DNA-PAINT. There is no trade-off in resolution, and the kinetics are very similar. The different DNA backbone and the resulting opposite circularization do not seem to impact the fluorogenic abilities of the imager strand. The properties of R-DNA imager sequences generally are well translatable to that of L-DNA imager probes.^14,26^ The strongly condensed total acquisition time of fluorogenic L-DNA-PAINT makes PAINT more user-friendly because it allows for higher throughput and at the same time reduces drift as a source of error in the final obtained data sets.

Besides the fast acquisition, L-DNA cannot bind to endogenous DNA and shows fewer unspecific epitope interactions. Our approach removes an important source of background binding in cells, especially relevant for single-molecule imaging. This small but significant reduction of the background is especially useful in challenging imaging conditions, such as for targets that are found deeper in biological samples or are surrounded by cellular DNA. We show that fluorogenic L-DNA-PAINT can yield strongly contrasted volumetric results for nuclear proteins. The reduction in the fluorescence background due to the fluorogenic nature of our imager probes combined with the reduced off-target binding of fluorogenic L-DNA-PAINT may also improve access to targets of low abundance or that can be only minimally labeled (e.g., by direct monoclonal antibody or nanobody).^14,27–30^ In these situations, on-target point accumulation above noise levels becomes more challenging, and fluorogenic L-DNA-PAINT shows promise.

Naturally, the absence of cross-hybridization between fluorogenic R- and L-DNA probes and their respective docking oligomers allows multiplexed imaging using the same nucleotide sequences in both probes. This enlarges the possibilities for multiplexing using fluorogenic probes, as has been shown for nonfluorogenic DNA-PAINT.^26^ This will, in the long term, improve the image quality in extensive multichannel acquisitions. In such experiments, simplicity and speed are key to achieving experimental throughput. To our knowledge, fluorogenic DNA-PAINT is one of the fastest DNA-PAINT methods currently available.^16,18^ In combination with exchange DNA-PAINT, it holds key advantages over handling-intensive approaches including several washes or enzymatic reactions.^31^ As we show here and others have demonstrated for nonfluorogenic DNA-PAINT,^26^ the use of L-DNA increases the range of possible DNA-PAINT probes, but the development of more specifically fluorogenic DNA-PAINT sequences would be beneficial. This would allow the acquisition of multichannel data from large multicellular areas in short amounts of time. Acquiring high-resolution data in this quantity will boost the identification and quantification of molecular complexes and may expose more rare biological events for investigation. We look forward to seeing how fluorogenic L-DNA-PAINT will help us understand the complex dynamics of cellular organization and function.

## Supplementary Material



## Data Availability

Data sets (localization files and confocal images) presented in the paper are accessible in the zenodo.org repository (DOI: 10.5281/zenodo.17699070). Extended data files are available upon request to the corresponding author(s) of the publication.

## Notes

Material and methods, fluorogenic probe attributes (Supporting Figure 1), estimation of the localization precision (Supporting Figure 2), photon counts and binding times (Supporting Figure 3), assessment of unspecific imager strand binding (Supporting Figure 4), and examples of volumetric fluorogenic DNA-PAINT (Supporting Figure 5) (PDF)
