# Supplementary material for: Accelerated Left-Handed DNA-PAINT Using Fluorogenic Probes
Source: Nano Lett. 2026 Apr 29;26(18):6026–33. doi: 10.1021/acs.nanolett.5c05973 (PMC13178128; doi:10.1021/acs.nanolett.5c05973)
Supplement: Supplementary file 1 [file nl5c05973_si_001.pdf]

Supporting Information

Accelerated Left-Handed DNA-PAINT Using Fluorogenic Probes

Bas van Bommel[1*] and Helge Ewers[1*]

1. Institut für Biochemie, Freie Universität Berlin, Thielallee 63, 14195 Berlin, Germany

bas.van.bommel@fu-berlin.de & helge.ewers@fu-berlin.de

* Corresponding authors

Content **Page**

Material and Methods 2 - 10

Supporting figures 1-5 12 - 17

Material and methods

Sample preparation

Cell culture

COS-7 cells (DSMZ – ACC60) and NRK52E-Septin-2-EGFP$^{EN/EN}$ [1] were cultured in either DMEM (*Thermo Fisher*) or Advanced DMEM (*Thermo Fisher*) supplemented with 2 mM L-glutamine (*Thermo Fisher*), 10% heat-inactivated fetal bovine serum and 1x Penicillin-Streptomycin (*Thermo Fisher*). Cells were split twice per week with 0.25% trypsin/EDTA (*Thermo Fisher*) and cultured to a maximum of 20 passages. Cultures were frequently tested for mycoplasma by DAPI staining and PCR.

The NRK52E-Septin-2-EGFP$^{EN/EN}$ was used for experimental data displayed in Figure 3 c-e and Supporting figure 5. All other experiments were conducted using COS-7 cells.

Immunocytochemistry

For immunocytochemistry (ICC) cells were cultured on plasma cleaned glass coverslips of #1.5 thickness.

For microtubule stainings (Figure 1, 2, 4a, Supporting figure 1, 2, 3), cells were extracted with 0.1% Triton-X-100 in PEM buffer (80 mM PIPES, 2 mM $MgCl_2$, 0.5 mM EGTA, 5% glycerol – pH 6.9 with KOH) for 30 s. Then cells were fixed with 4% formaldehyde and 0.25% glutaraldehyde for 10 min at 37 °C. Other samples (Figure 4d) were fixed with 4% PFA /4% sucrose in PBS, equally for 10 min at 37 °C. Cells were washed 3 times with PBS after fixation, then permeabilized with 0.2% Triton-X-100 in PBS for 10 min. Afterwards, cells were washed once with PBS and incubated in blocking buffer (BB – 10% horse serum, 0.1% Triton-X-100 in PBS) for 1 h at RT (room temperature). Subsequently cells were blocked with Image-iT FX Signal Enhancer (*Thermo Fisher*) for 30 minutes at RT. Then cells were incubated with primary antibody in BB overnight at 4 °C. Next, cells were washed 3 times with PBS and incubated with secondary antibody in BB overnight at 4 °C. The cells were washed 3 times with PBS before imaging in DNA-PAINT buffer (see below). For confocal examples, coverslips were mounted with Fluoromount-G (*Thermo Fisher*) onto glass slides.

For volumetric DNA-PAINT (Figure 3e, Supporting figure 5), samples were fixed with 4% PFA /4% sucrose in PBS prewarmed to 37 °C for 10 min. After which the samples were washed 3 times with PBS, then permeabilized with 0.2% Triton-X-100 in PBS for 10 min. Subsequently, the samples were washed once with PBS and blocked for 1 hour at RT with BB. Samples were incubated with primary antibody overnight at 4 °C in BB. The following day, samples were washed 3 times with PBS (10 min each) and incubated with secondary antibody overnight at 4 °C in BB. Samples were washed 3 times with PBS, before microscopy was conducted in DNA-PAINT buffer. All PBS washing steps were conducted at RT.

For the analysis of unspecific imager binding (Figure 3a, b), samples were fixed with 4% PFA /4% sucrose in PBS prewarmed to 37 °C for 10 min. After which the samples were washed 3 times with PBS, then permeabilized with 0.2% Triton-X-100 in PBS for 10 min. Subsequently, the samples were washed once with PBS and blocked overnight at 4 °C in BB. The following day, the samples were washed 3 times with PBS at RT, before imaging in DNA-PAINT buffer. All PBS washing steps were conducted at RT.

For the analysis of unspecific antibody binding, cells were fixed using 4 different methods; 1) with 4% PFA /4% sucrose in PBS prewarmed to 37 °C for 10 min, 2) with 4% PFA /4% sucrose /0.25% glutaraldehyde in PBS prewarmed to 37 °C for 10 min, 3) with pre-cooled (-20 °C) 100% methanol for 10 min, and 4) glyoxal fixation with 3% v/v glyoxal, 20% ethanol, 0.75% acetic acid, adjusted to pH 5 with NaOH for 30 min on ice followed by 30 min at RT as described in Richter et al., 2018[2]. All samples were subsequently washed 3 times with PBS, then permeabilized with 0.2% Triton-X-100 in PBS for 10 min. Washed once with PBS, and blocked for 1 h at RT with BB. Secondary antibodies (goat anti-rabbit conjugated with docking strand F1R/L at 1:250 from a ~1 mg/ml stock and goat anti-rabbit Alexa Fluor 568 at 1:500 from a ~2 mg/ml stock) were incubated overnight at 4 °C in BB. The next day, the samples were washed 3 times (10 min each) with PBS. Samples were incubated with donkey anti-goat Alexa Fluor 647 (1:250 from a ~2 mg/ml stock) for 2 h at RT in BB. After 2 h of incubation, the samples were washed twice in PBS (10 min each), followed by 1 wash with Hoechst 33342 at 10 µg/ml in PBS for 10 min, followed by two washes with PBS each 10 min. All PBS washing steps were conducted at RT. The samples were imaged with confocal microscopy at on the same day in DNA-PAINT buffer, as done for DNA-PAINT imaging. Buffer exchange to DNA-PAINT buffer was conducted when mounting the samples in the imaging chamber.

Preparation of secondary antibodies for DNA-PAINT

Docking oligomers were conjugated to secondary antibodies as described previously[3]. Briefly, antibodies were conjugated with DBCO-NHS (Sigma-Aldrich, 761524-5 MG) and subsequently coupled via copper-free click chemistry with azide-modified docking oligos.

Table 1: Primary antibodies used for this study

Target	Host species	Clonality	Dilution	Supplier	Product nr.
α - tubulin	Mouse	Monoclonal	1:250-500	Sigma Aldrich	T5168
β - tubulin	Mouse	Monoclonal	1:250-500	Sigma Aldrich	T5293
Acetyl - α - tubulin	Rabbit	Monoclonal	1:250-500	Invitrogen	32-2700
Lamin B1	Rabbit	Polyclonal	1:250	Abcam	ab16048
Ki67 (for 2-color imaging)	Mouse	Monoclonal	1:250	BD Pharmingen	556003
Ki67 (for 3D imaging)	Rat	Monoclonal	1:250	Thermo Fisher /eBioscience	53-5698-82

Table 2: Secondary antibodies used for this study

Target	Host species	Clonality	Dilution	Conjugate	Supplier	Product nr.
Mouse	Donkey	Polyclonal	1:250	DNA	Jackson ImmunoResearch	715-005-150
Rabbit	Donkey	Polyclonal	1:250	DNA	Jackson ImmunoResearch	711-005-152
Rabbit	Goat	Polyclonal	1:250	DNA	Jackson ImmunoResearch	111-005-003
Rat	Donkey	Polyclonal	1:250	DNA	Jackson ImmunoResearch	712-005-150
Rabbit	Goat	Polyclonal	1:250	Alexa Fluor 488	Invitrogen	A11034
Mouse	Goat	Polyclonal	1:250	Alexa Fluor 568	Invitrogen	A11004
Rabbit	Goat	Polyclonal	1:500	Alexa Fluor 568	Invitrogen	A11036
Goat	Donkey	Polyclonal	1:250	Alexa Fluor 647	Invitrogen	A21447

Table 3: DNA strands used for this study

Identifier	Sequence 5'to 3'	Synthesized by
LP3	GTAATGAAGA/ Atto655	Biomers
LB3	Biotin /TTTCTTCATTA	Biomers
F - imager seq. 1 - L	Cy3B /AGAAGTAATGTGGAA/ BHQ3	Biomers
F - docking seq. 1 -L	CCTTCAACATATCCTCTAC/ Azide	Biomers
F - imager seq. 1 -R	Cy3B /AGAAGTAATGTGGAA/ BHQ3	Integrated DNA Technologies
F - docking seq. 1 -R	CCTTCAACATATCCTCTAC/ Azide	Integrated DNA Technologies / Biomers
F - imager seq. 2 -R	Atto643 /AAGAAGTAAAGGGAG/ IABkFQ	Integrated DNA Technologies
F - docking seq. 2 -R	CCTCGCTGAACCCCTTA/ Azide	Integrated DNA Technologies
Full complementary for F – imager seq. 1 - L	TTCCACATTACTTCT	Biomers
Full complementary for F – imager seq. 1 - R	TTCCACATTACTTCT	Microsynth
Full complementary for F – imager seq. 2 - R	CTCCCTTTACTTCTT	Microsynth

Microscopy

Confocal microscopy

Confocal images were acquired on a Yokogawa X1 spinning-disk microscope. The confocal unit was attached to a Nikon Ti1 microscope body fitted with a 100x 1.49 NA TIRF objective (*Nikon*). Excitation was provided by 405 nm, 488 nm and 561 nm excitation lasers coupled via a single mode polarization-maintaining optical fiber. Excitation and emission light were split via a quadband dichroic mirror in the confocal unit. Emission light was additionally filtered with a filter wheel equipped with emission filters: ET460/50m (DAPI), ET525/50m (Alexa Fluor 488) and ET609/54m (Alexa Fluor 568). Images were acquired on a water-cooled PCO.Edge 4.2 bi sCMOS camera with a sample pixel size of 65 nm. Image stacks were obtained with a step size of 0.2 µm. All microscope components were controlled by

VisiView (*Visitron systems*). Confocal images throughout the manuscript are presented as maximum intensity projections (MIP).

Confocal images for the quantification of unspecific antibody binding were acquired on a Yokogawa X1 spinning-disk microscope. The confocal unit was attached to an Olympus IX71 microscope body fitted with a 60x 1.42 NA objective (*Olympus*). Excitation was provided with 405 nm and 635 nm lasers (Gataca Systems) coupled via a single mode polarization-maintaining optical fiber. Excitation and emission light were split via a quadband dichroic mirror in the confocal unit. Emission light of only Alexa Fluor 647 was filtered additionally with a specific emission filter (700/75 ET). Images were acquired on an air-cooled Hamamatsu ORCA Flash 4.0LT Plus sCMOS camera, which had a sample pixel size of 90 nm. Image stacks were obtained with a step size of 0.3 µm using a piezo mounted at the objective. The microscope was controlled using MetaMorph software. Confocal images throughout the manuscript are presented as MIP.

SMLM microscopy

Images were acquired with a Vutara 352 microscope system (*Bruker*), configured in bi-plane mode. The system was equipped with a 60x objective (Olympus APON60XOTIRF – 1.49 NA - Oil) resulting in a final pixel size of 96.63 nm. Fluorophores were excited with a 560 nm (Cy3B – fluorogenic R- and L-DNA-PAINT probes) and 640 nm laser (Atto643/ Atto655 fluorogenic/ non-fluorogenic R- and L-DNA-PAINT probes); laser power was controlled with an acousto-optic tunable filter. Samples were illuminated in TIRF mode or with a highly inclined illumination angle. A pentaband dichroic mirror (405/488/561/640/750) was used to split the excitation and emission light, and a second pentaband emission filter was used to clean up emission light from residual excitation. Emission light was subsequently filtered for the imaged channel via emission filters located in a filter wheel in front of the camera (‘Vutara Orange single bandpass’ ~600/50 nm for Cy3B, ‘Vutara Red single bandpass’ ~ 692.5/85 nm). Images were recorded on a water-cooled Hamamatsu Orca Flash 4.0 with a sample pixel size of 98.69 nm.

For imaging, fluorogenic imager probes were diluted in DNA-PAINT buffer (1x PBS, supplemented with additional 500 mM NaCl, 20 mM Na_2SO_3 and 1 mM Trolox, ~pH 7.4) at a concentration of 4 (F2R) – 10 (F1R/L) nM. Trolox (Santa Cruz Biotechnology, sc-200810) was added from a 50 mM stock dilution in DMSO. Raw images, of 40 µm x 40 µm, were acquired with 20 ms exposure at 50 Hz. Each acquisition consisted of 30.000 images.

For non-fluorogenic L-DNA PAINT (figure 1c) imager strands were dissolved in imaging buffer (1x PBS with 500 mM NaCl) at a concentration of 1 nM. Raw images, of 40 µm x 40 µm, were acquired with 300 ms exposure at 3.33 Hz. The acquisition contained ~12.000 raw frames, the reconstruction was created from a subset of 10.000 images.

For imaging of unspecific imager strand binding (figure 3a), imager strands were diluted in DNA-PAINT buffer at a concentration of 10 nM each. Raw images, of 40 µm x 40 µm, were acquired with 20 ms exposure at 50 Hz. Each acquisition consisted of 30.000 images.

Volumetric DNA-PAINT of the nucleus was conducted in a widefield illumination scheme. 561 nm laser light was coupled to the microscope via a squared core optical fiber, shaken to create an even illumination. Volumetric data was acquired using the bi-plane configuration with the focusing planes approximately ~1 µm apart. Calibration was conducted by taking z-stacks of 0.1 µm fluorescent TetraSpeck Mircospheres (*Thermo Fisher*) in the Vutara SRX software. Imager strands were added in

a concentration of 10 nM for Lamin B1 and 5 nM for Ki67. The dense packing of Ki67 in clusters required lowering the imager strand concentration to be able to obtain singular PSFs. Raw images, of 40 µm x 40 µm, were acquired with 40 ms exposure at 25 Hz. Z-stepping was conducted with a step size of 0.5 µm, 10.000 images were acquired at each step.

Fluorophores were localized with the Vutara software (*Bruker*). Molecules were fitted using maximum likelihood estimation Gaussian fitting. Particle linking was conducted for 4 frames for volumetric DNA-PAINT and 6-8 frames for L-DNA-PAINT and fluorogenic R-/L-DNA-PAINT with a maximal offset of 2 pixels (~180 nm). No or max particle linking was conducted when that was required for the specific analysis. The obtained coordinates and statistics of the fitted molecules were exported in .CSV format and imported into Matlab. Here we performed drift correction in 3D by redundant cross-correlation based on scripts from Wang et al., 2014[4]. Ki67 (dual-color) data was drift corrected using AIM[5]. Localizations with a precision estimate (*Cramér-Rao Lower Bound)* >20 nm XY or >60 nm Z were excluded. Super-resolved reconstructions were made by plotting the localizations in 2D as accumulated Gaussians with sigmas corresponding to 12 nm FWHM. Reconstructions were rendered with a pixel size of 20 nm for overview images, insets are rendered at a pixel size of 4 nm. Volumetric reconstructions were color coded for Z. For visualization contrast normalization was applied, for direct comparisons images were scaled with identical brightness and contrast settings.

Data analysis

Spectrum analysis of DNA-PAINT probes

The spectra and fluorogenic properties of the DNA-PAINT probes were analyzed using a Tecan Spark plate reader. Imager strands (F1R, F1L and F2R) were diluted to a concentration of 1 µM and 0.1 µM in DNA-PAINT buffer. Measurements of the 1 µM concentration were used for the final analysis, as these showed highest signal-to-noise ratios. There was no indication that the spectra were influenced by concentration. Bound state was measured by the addition of 10 µM fully complementary docking strands (no DNA mismatches, dissolved in H_2O), equal volumes of H_2O were added to the unbound state. In addition, we measured 'blank' DNA-PAINT buffer (with additional H_2O for balancing concentrations) for background subtraction/blanking. Solutions were measured in a black (emission) and transparent (absorption) 96-well plate (Corning), in volumes of 60 µl per well. Detector gain settings were set to 125, temperature was controlled in a range from 20 to 21 °C (typical ~20.5 °C during measurement). Absorbance was measured from 400 to 900 nm, 1 flash with a step size of 2 nm. For emission measurements, Cy3B was excited at 510 nm and Atto643 at 580 nm with an excitation bandwidth of 10 nm for each. Emission was recorded for Cy3B from 535 to 750 nm, for Atto643 from 620 to 900 nm. Emission was measured with a bandwidth of 10 nm, a step size of 2 nm, 30 flashes followed by an integration time of 40 µs. All experimental groups were measured once from 5 different wells (repeats).

Results were exported in Excel format, analysis was conducted with Matlab scripts. Absorption measurement in a few wells showed axial shift. This axial shift was first corrected by translating the full absorption spectra to the median values of each group based on the region 750 – 900 nm (in which fluorophores did not absorb). After translation, the average for DNA-PAINT buffer (blank) was computed and subtracted from each absorption spectra. Next, the absorption spectra for each group were averaged and normalized (0-100) for each unbound/bound sequence combination relative to the emission spectra.

For emission analysis, measurements of blank DNA-PAINT buffer were averaged and subtracted from each emission spectrum. Next, the emission spectra were averaged and normalized (0-100) for each unbound/bound sequence combination. The baseline subtracted averaged and normalized absorption and emission spectra were exported in an Excel file, final graphs were created in GraphPad Prism. Reference absorption spectra for singular Cy3B and Atto643 (dotted grey in figure) were obtained from fpbase.org[6].

Unspecific antibody binding analysis

For each of the 4 different fixation methods and tested antibodies, 20 image stacks were transformed to a maximum intensity projection using a FIJI macro. A next FIJI macro opened and processed the maximum intensity projection one-by-one in a loop. First, channel 2 (Hoechst 33342) was duplicated, and the duplicate was processed using a sigma 2.0 Gaussian blur. The obtained image was used to apply a threshold to create a ROI selection for the nuclei. The obtained ROI selection was 'split' to create individual ROIs for the nuclei. A >50 μm^2 filter was applied to remove noise/small ROIs. The obtained ROIs were used to measure 'mean' intensity for each ROI in the 647 (Antibody) and 405 (Hoechst 33342) channel from the unprocessed maximum intensity projection. The ratio (mean antibody/mean Hoechst 33342) was calculated for each ROI (nucleus). These ratios were averaged to one value per acquisition (data point). The data points were plotted in a bar graph with SEM using GraphPad Prism.

Microtubule FWHM

Microtubule cross-sections were made using the 'Plot profile' function in FIJI/ImageJ. For FWHM analysis, the intensity profile was fitted with a Gaussian curve ($y = a + (b-a)*exp(-(x-c)*(x-c)/(2*d*d))$) in FIJI[7]. The standard deviation (d) was used to compute the FWHM in Excel. For each acquisition/reconstruction two profiles were analyzed. The obtained values were used to make a plot in GraphPad Prism.

Fourier ring correlation (FRC) estimates

FRC measurements were performed in FIJI/ImageJ with a three sigma threshold[8]. The images for the measurement were prepared in Matlab, by making two reconstructions per acquisitions. For this, the localizations were split by row number; even vs. odd. The localization table was obtained after particle linking with a maximum of 6-frames. The final images were rendered with a pixel size of 10 nm, localizations were plotted as a Gaussian with a FWHM of 12 nm. Results were plotted in a bar graph, and line profiles were made in GraphPad Prism.

Binding time analysis

Binding time was analyzed based on the particle linking information provided by the Vutara software. For this analysis, localizations were linked for infinite number of frames (instead of max 8 frames for reconstructions). Maximal displacement was set to 2 pixels (~ 180 nm). Binding time was estimated based on the number of frame accumulations for each localization. In Matlab, the accumulation values were counted and sorted into bins. The bins were normalized from 0-100 percent for each acquisition. This allowed comparison between acquisitions independent of the number of total localizations (typically > 1.5 million). The binned, normalized values were plotted in GraphPad Prism.

Photon count analysis

Photon count was analyzed by plotting the 'psf-photon-count' from the Vutara localization table. In other words, the photons associated with the PSF. No particle linking was applied for this analysis. The values for all the localizations were binned and normalized per acquisition in Matlab. The normalized values of each acquisition were combined for the final plots. Plots were made in GraphPad Prism.

Localization precision estimate Cramér-Rao Lower Bound (CRLB)

Localization precision was estimated using the CRLB criteria and computed by the Vutara software after fitting[9]. The values were obtained from the localization table, binned and normalized per acquisition in Matlab. Particle linking was applied for 8 frames. The normalized values of each acquisition were combined for the final plots. Plots were made in GraphPad Prism.

Nearest Neighbor in Adjacent frames (NeNA) analysis

The NeNA analysis was conducted with non-linked localization tables. The pairwise distance from closest neighbors in adjacent frames were computed in Matlab using the function 'pdist'. The obtained displacements were sorted into histograms with a bin size of 1 nm and a maximum bin number of 300. The histograms were normalized from 0-100 percent, and histograms from multiple acquisitions were averaged into a single histogram. The obtained histogram was fitted with the *P(d ij)* function as described previously[10,11]. Plots were made in GraphPad Prism.

Pixel-based Pearson's correlation

Pixel-based correlation was performed based on reconstructions with a pixel size of 20 nm and 3x binning, resulting in a final pixel size of 60 nm. Input images are displayed in the corresponding figure. Intensities were normalized and plotted in X (fluorogenic R-DNA-PAINT) and Y (fluorogenic L-DNA-PAINT) axes. Plots are displayed as density plots after a Gaussian blur with sigma 5. The 0/0 pixel value, which dominated the plot, was clipped. All analysis and plotting were performed in Matlab.

Table 4: Software used for analysis and visualization

Software	version	Manufacturer
Vutara SRX	7.0.06	Bruker, Bellerica, Massachusetts
FIJI	ImageJ 1.53q	Schindelin et al., 2012[7]
Matlab	R2024a/b	The MathWorks Inc., Natick, Massachusetts
GraphPad Prism	10.4.2	GraphPad Software, Boston, Massachusetts
Affinity Designer 2	2.6.3	Affinity, Nottingham, Nottinghamshire

Image presentation

Confocal images and (Matlab) obtained SMLM reconstructions were further processed in FIJI/ImageJ. Here the images were cropped if needed, and assigned a lookup table. Graphs and statistics were completed in GraphPad Prism. Figure composition was performed in Affinity Designer 2.

Information on replicates

Single channel microtubule datasets for the different fluorogenic probes were acquired in 2 independent experiments (N = 2). With a total for 10 acquisitions for R- fluorogenic sequence 1, 13 acquisitions for fluorogenic L-DNA sequence 1, and 10 for fluorogenic R-DNA sequence 2 (n = 10, n = 13, n = 10). One fluorogenic L-DNA sequence 1 acquisition was excluded from the analyses due to extensive drift that could not be adequately corrected. Non-fluorogenic L-DNA-PAINT data was recorded in one experiment twice (N = 1, n = 2). Absorption and emission from unbound and bound imager strands were measured in 1 experiment with 5 individual wells per condition with a plate reader. Background binding of the imager strands was estimated from 2 independent experiments with 5 acquisitions for each of the fluorogenic probes (N = 2, n = 10). Background binding of the conjugated antibodies was quantified from 2 independent experiments with 10 acquisitions each per experimental group (N = 2, n = 20). Volumetric DNA-PAINT experiments for Lamin B1 and Ki67 were each conducted once, results of the acquisitions are presented in the figures and supporting information. Dual channel microtubule datasets (tub. /acetyl. tub.) were obtained from one experiment with 7 acquisitions (N = 1, n = 7). One representative acquisition is displayed in this publication and more extensively analyzed. Dual channel datasets from the nucleus were obtained in one experiment with 5 acquisitions (N = 1, n = 5). One representative acquisition is displayed in this publication and more extensively analyzed.

References:

(1) Zehtabian, A.; Müller, P. M.; Goisser, M.; Obendorf, L.; Jänisch, L.; Hümpfer, N.; Rentsch, J.; Ewers, H. Precise Measurement of Nanoscopic Septin Ring Structures with Deep Learning-Assisted Quantitative Superresolution Microscopy. *Mol. Biol. Cell* **2022**, *33* (8), 1–13. https://doi.org/10.1091/mbc.E22-02-0039.

(2) Richter, K. N.; Revelo, N. H.; Seitz, K. J.; Helm, M. S.; Sarkar, D.; Saleeb, R. S.; Este, E. D.; Eberle, J.; Wagner, E.; Vogl, C.; Lazaro, D. F.; Richter, F.; Coy-vergara, J.; Coceano, G.; Boyden, E. S.; Duncan, R. R.; Hell, S. W.; Lauterbach, M. A.; Lehnart, S. E.; Moser, T.; Outeiro, T. F. Glyoxal as an Alternative Fixative to Formaldehyde in Immunostaining and Super-Resolution Microscopy. *EMBO J.* **2018**, *37* (1), 139–159. https://doi.org/10.15252/embj.201695709.

(3) Schnitzbauer, J.; Strauss, M. T.; Schlichthaerle, T.; Schueder, F.; Jungmann, R. Super-Resolution Microscopy with DNA-PAINT. *Nat. Protoc.* **2017**, *12* (6), 1198–1228. https://doi.org/10.1038/nprot.2017.024.

(4) Wang, Y.; Schnitzbauer, J.; Hu, Z.; Li, X.; Cheng, Y.; Huang, Z.-L.; Huang, B. Localization Events-Based Sample Drift Correction for Localization Microscopy with Redundant Cross-Correlation Algorithm. *Opt. Express* **2014**, *22* (13), 15982. https://doi.org/10.1364/oe.22.015982.

(5) Ma, H.; Chen, M.; Nguyen, P.; Liu, Y. Toward Drift-Free High-Throughput Nanoscopy through Adaptive Intersection Maximization. *Sci. Adv.* **2024**, *10* (21), 1–11. https://doi.org/10.1126/sciadv.adm7765.

(6) Lambert, T. J. FPbase: A Community-Editable Fluorescent Protein Database. *Nat. Methods* **2019**, *16* (4), 277–278. https://doi.org/10.1038/s41592-019-0352-8.

(7) Schindelin, J.; Arganda-Carreras, I.; Frise, E.; Kaynig, V.; Longair, M.; Pietzsch, T.; Preibisch, S.; Rueden, C.; Saalfeld, S.; Schmid, B.; Tinevez, J. Y.; White, D. J.; Hartenstein, V.; Eliceiri, K.; Tomancak, P.; Cardona, A. Fiji: An Open-Source Platform for Biological-Image Analysis. *Nat. Methods* **2012**, *9* (7), 676–682. https://doi.org/10.1038/nmeth.2019.

(8) Nieuwenhuizen, R. P. J.; Lidke, K. A.; Bates, M.; Puig, D. L.; Grünwald, D.; Stallinga, S.; Rieger, B. Measuring Image Resolution in Optical Nanoscopy. *Nat. Methods* **2013**, *10* (6), 557–562. https://doi.org/10.1038/nmeth.2448.

(9) Ober, R. J.; Ram, S.; Ward, E. S. Localization Accuracy in Single-Molecule Microscopy. *Biophys. J.* **2004**, *86* (2), 1185–1200. https://doi.org/10.1016/S0006-3495(04)74193-4.

(10) Endesfelder, U.; Malkusch, S.; Fricke, F.; Heilemann, M. A Simple Method to Estimate the Average Localization Precision of a Single-Molecule Localization Microscopy Experiment. *Histochem. Cell Biol.* **2014**, *141* (6), 629–638. https://doi.org/10.1007/s00418-014-1192-3.

(11) Martens, K. J. A.; Turkowyd, B.; Endesfelder, U. Raw Data to Results: A Hands-On Introduction and Overview of Computational Analysis for Single-Molecule Localization Microscopy. *Front. Bioinforma.* **2021**, *1* (February), 1–19. https://doi.org/10.3389/fbinf.2021.817254.

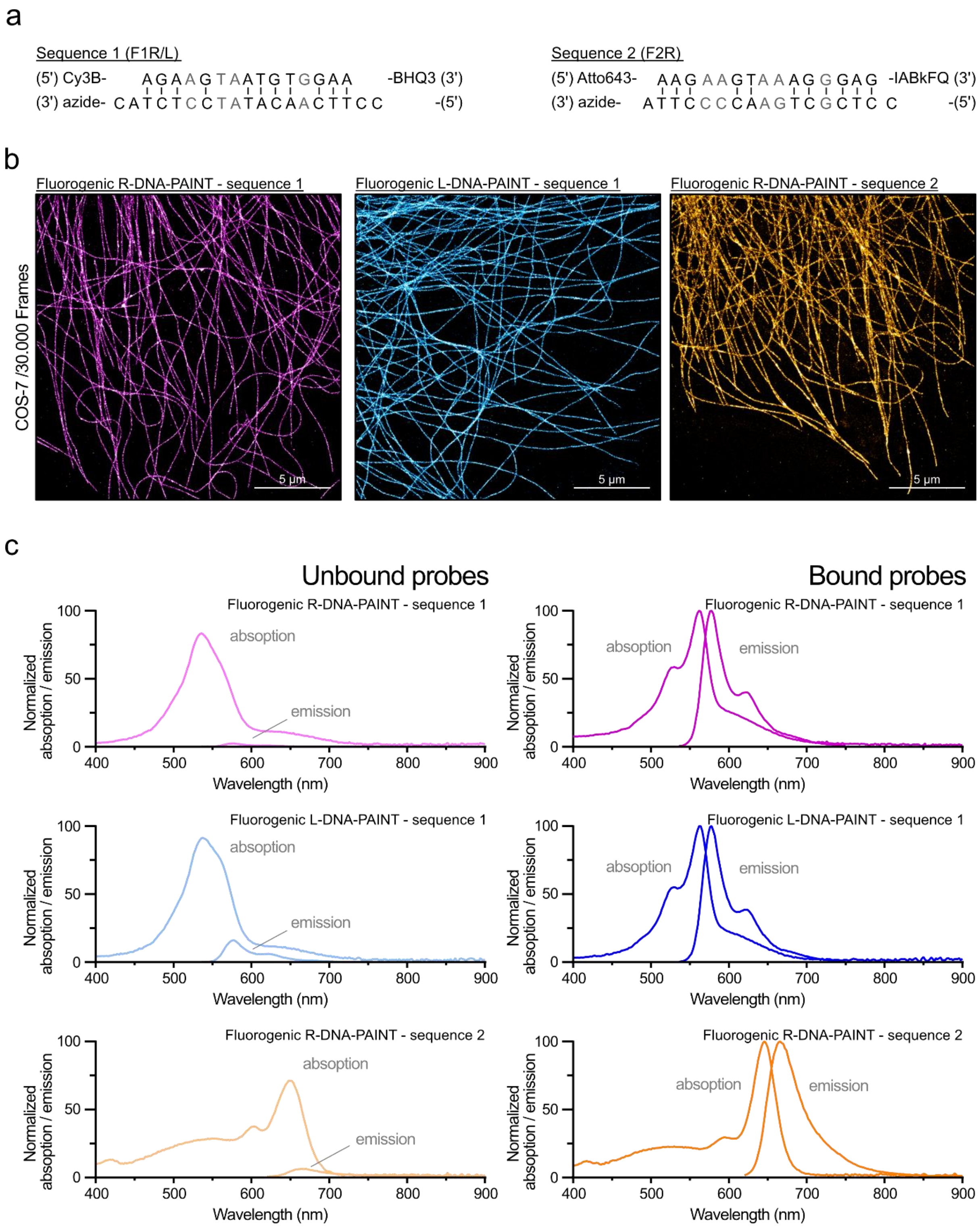

Supporting figure 1. Probe attributes of fluorogenic R- and L-DNA-PAINT.

a) DNA-PAINT imager and docking sequences as used in this study. Matching and mismatching bases are indicated.

b) DNA-PAINT acquisitions of microtubules in COS-7 cells using the indicated probes acquired with TIRF excitation.

c) Absorption and emission spectra of fluorogenic probes in unbound and bound state. Spectra obtained from the plate reader are normalized between 0 and 100 for absorption and emission.

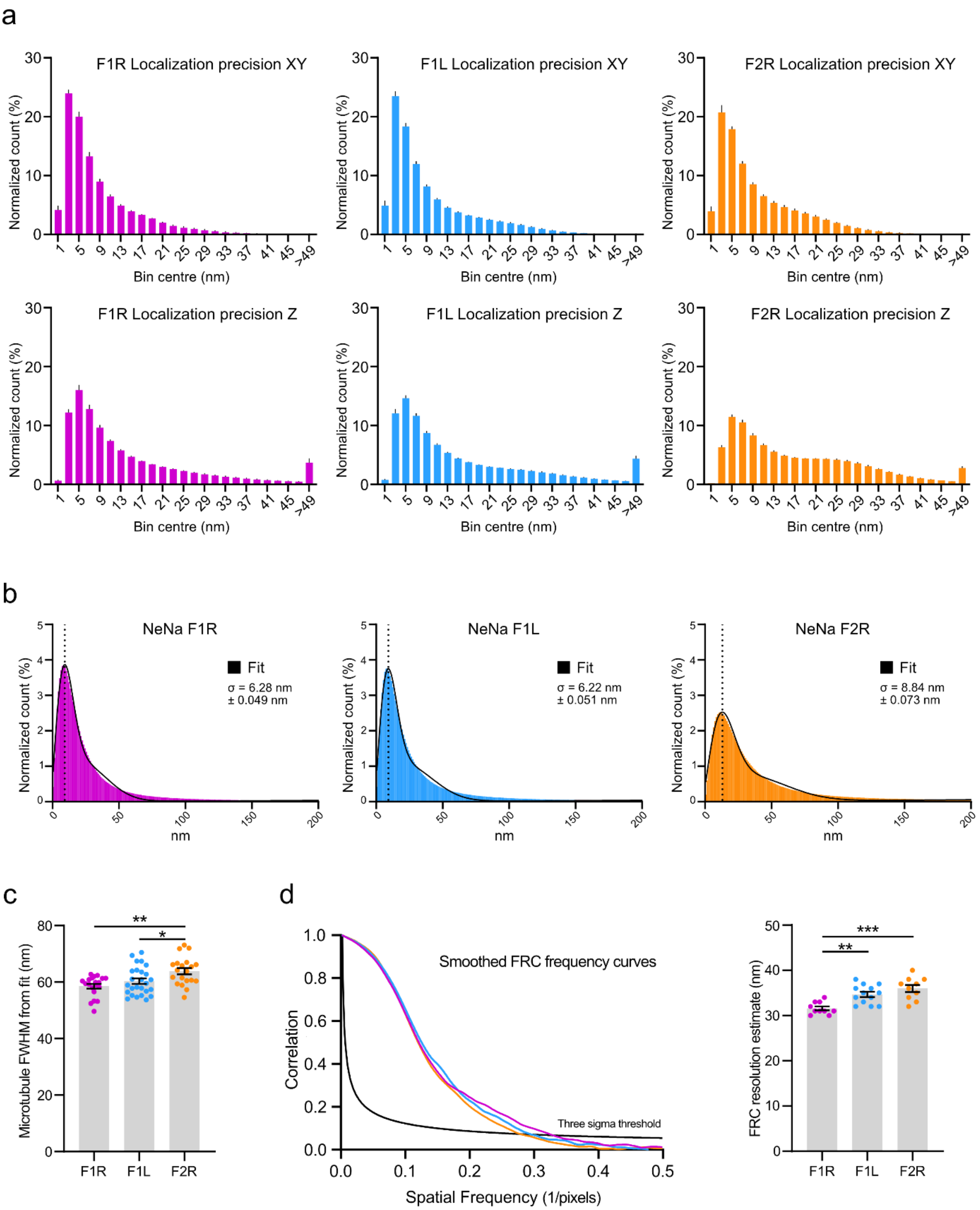

Supporting figure 2. Estimation of localization precision for fluorogenic DNA-PAINT probes.

a) Distribution of localization precision estimated by Cramér-Rao Lower Bound. Distribution of localization precision in XY (top) and Z (bottom) for different fluorogenic probes. Bars show average with SEM, error bars are plotted upward only.

b) Estimate of localization precision by nearest neighbor (NeNA) analysis for different fluorogenic DNA-PAINT probes. Histogram is an average of multiple acquisitions, fitted with a *P(d ij)* function (see methods for details). Fluorogenic R- and L-DNA-PAINT probes show a very similar precision.

c) Resolution estimates by FWHM measurements from microtubule cross-sections (fit) for fluorogenic DNA-PAINT probes. Two measurements per acquisition/reconstruction. Error bars show SEM (one-way ANOVA with Tukey's post hoc test for multiple comparisons. F1R vs F2R: $p = 0.002$; F1L vs F2R, $p = 0.03$).

d) Example of a Fourier ring correlation (FRC) measurement for fluorogenic R- and L-DNA-PAINT acquisitions.

e) Quantification of FRC for multiple fluorogenic R- and L-DNA-PAINT acquisitions. Bars show average with SEM (one-way ANOVA with Tukey's post hoc test for multiple comparisons: F1R vs F1L, $p = 0.003$, F1R vs F2R, $p < 0.001$).

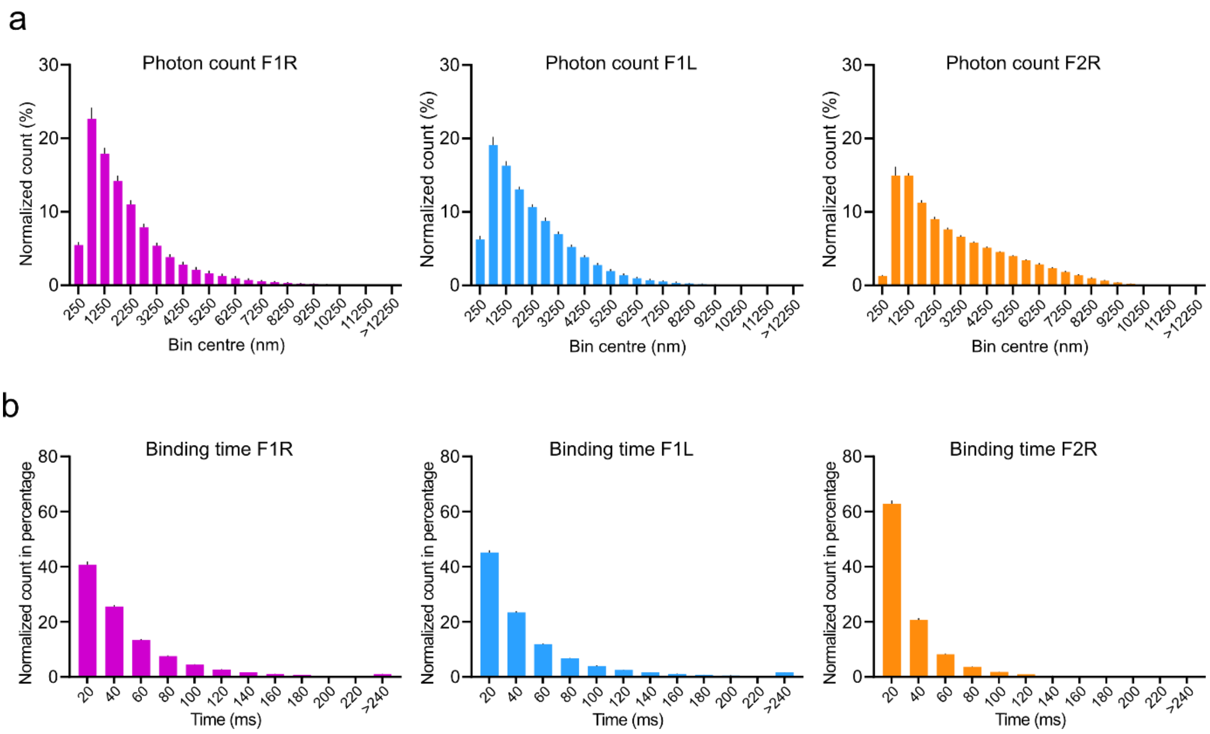

Supporting figure 3. Measured photon counts and binding times for fluorogenic DNA-PAINT probes.

a) Distribution of estimated photon counts (from Gaussian fits) for fluorogenic DNA-PAINT probes. Bars show average with SEM, error bars are plotted upward only.

b) Distribution of estimated binding times for fluorogenic DNA-PAINT probes (see methods for details). Data was acquired with a 20 ms exposure time per frame. Bars show average with SEM, error bars are plotted upward only.

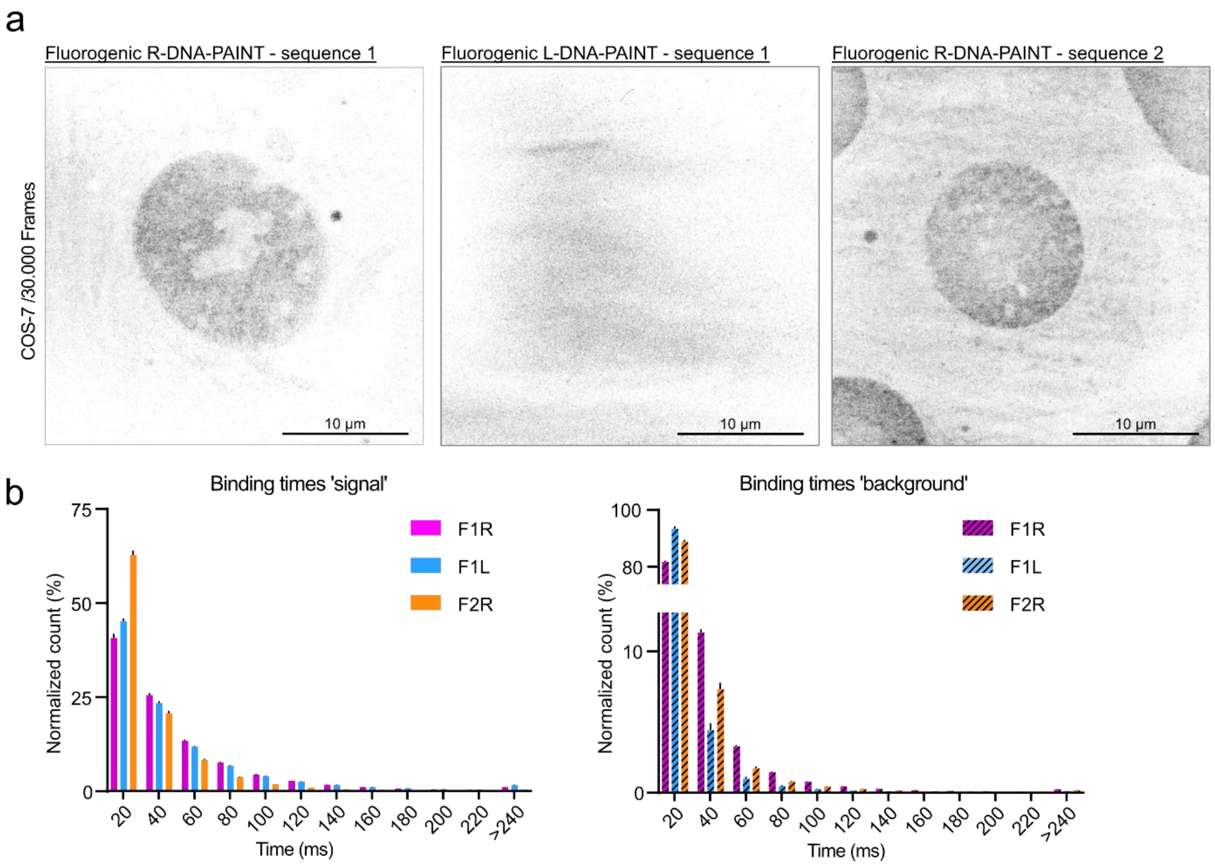

Supporting figure 4. Further assessment of unspecific imager strand binding.

a) Example of background patterns as a result of unspecific binding of the imager probes. Fluorogenic R-DNA-PAINT shows background binding in the nucleus, while fluorogenic L-DNA probes solely include illumination/camera noise. Note that there is also a difference between the tested fluorogenic R-DNA-PAINT sequences.

b) Binding times recorded from microtubule samples (left). Data was recorded with 20 ms exposure time per frame. Right: binding times of noise patterns as illustrated in Supporting figure 4a, fluorogenic L-DNA-PAINT shows fewer events that persist across multiple frames. Bars show average of multiple acquisitions with SEM, error bars are plotted upward only (Binding times 'signal': repeated-measures two-way ANOVA with Šidák's test for multiple comparisons: 20 – 60 ms, F1R vs F1L vs F2R, $p < 0.01$; 80-120 ms, F1R vs F2R and F1L vs F2R, $p < 0.01$. Binding times 'background': repeated measures two-way ANOVA with Šidák's test for multiple comparisons: 20 – 40 ms, F1R vs F1L vs F2R $p < 0.001$; 60 ms, F1R vs F1L and F1R vs F2R, $p < 0.001$; 80 ms, $p < 0.01$).

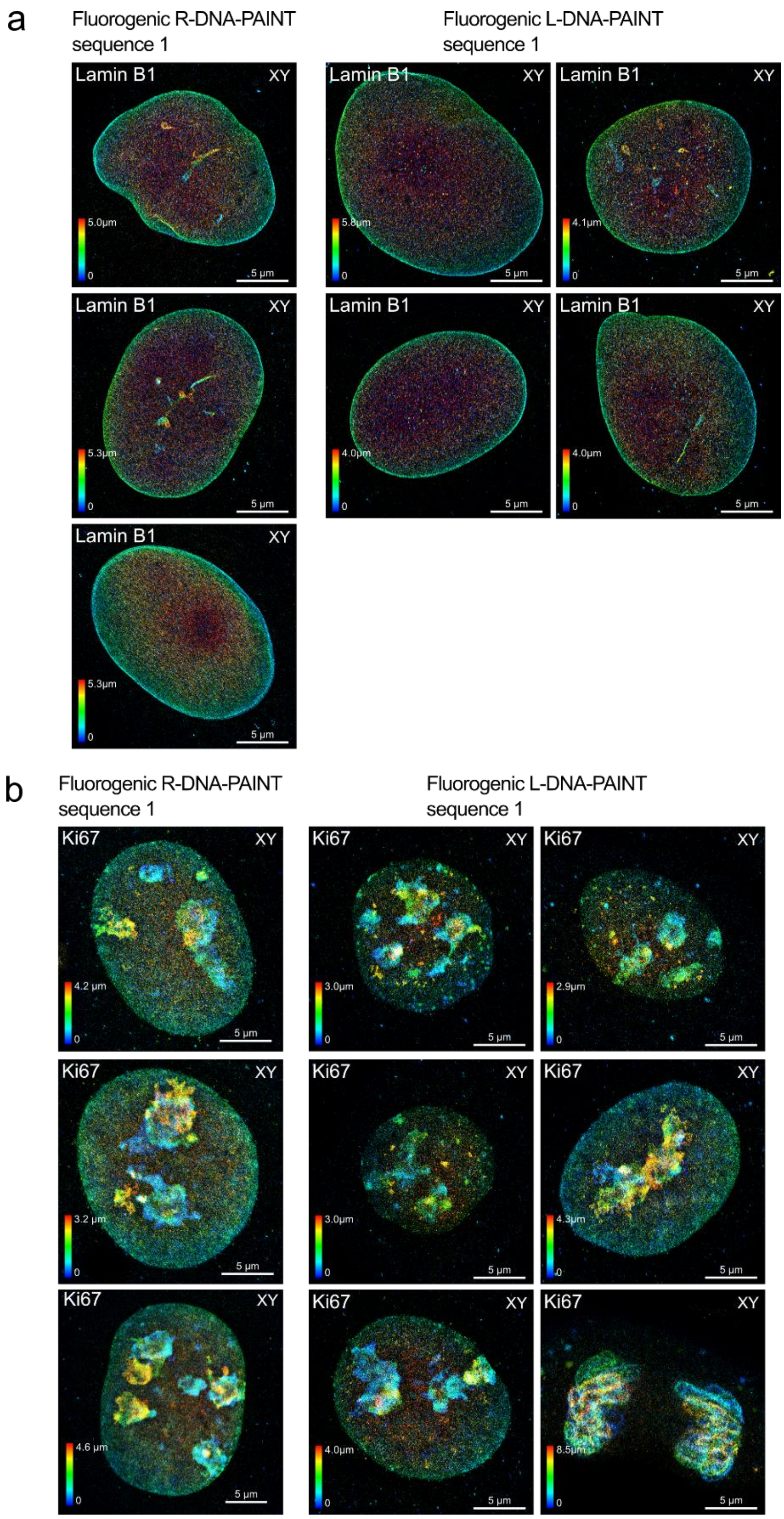

Supporting figure 5. Further examples of volumetric fluorogenic DNA-PAINT.

a) Examples of volumetric fluorogenic R- and L-DNA-PAINT for Lamin B1. Reconstructions are color-coded for Z.

b) Examples of volumetric fluorogenic R- and L-DNA-PAINT for Ki67. Reconstructions are color-coded for Z.
